# Four Gynecological Facts Not Usually Mentioned in Text-Books

**Published:** 1880-02-20

**Authors:** O. E. Herrick


### *FOUR GYNECOLOGICAL FACTS NOT USUALLY MENTIONED IN TEXT-BOOKS.*

BY O. E. HERRICK, M.D.

(*a*) *How to Make a Vaginal Examination.*—We often hear physicians tell of difficulty in reaching the os in making digital examinations, and indeed there *is* often difficulty in doing so, with the patient on her back, or side, with her face towards the operator; especially if the person making the examination has short fingers. That difficulty is easily overcome if the patient is placed upon her side, (either right or left) with one or both knees drawn up, while the operator sits behind her and makes the examination from behind. In this position the os is easily accessible, and can be reached in from 2½ to 3 inches from the vulva.

(*b*) *How to Make an Ocular Inspection of the Os Uteri Without a Speculum.*—Put the patient in Sims' position, and with the two front fingers of the right hand retract the posterior vaginal walls exactly as with a Sims' speculum, when a good view of the os can be obtained; oftentimes one wishes to make a specular examination when he does not have his speculum with him; under such circumstances the fingers will many times answer every purpose; the air enters the vagina and distends it just the same as when a Sims' speculum is used. In this way the posterior wall of the vagina is retracted somewhat, which brings the os uteri considerably nearer the vulva than it otherwise would be, and if the posterior wall was *not* retracted, it would still be more accessible from behind; this is easily seen when one remembers that the direction of the vagina is *upward* and *backward*. With a woman upon her back, her os uteri is more inaccessible than in any other position she could get into, and yet that is the position almost always chosen for both digital and ocular examinations. Many men who have practiced medicine thirty or forty years still put their patients upon their backs to examine them; indeed I do not remember to have seen over three or four men who are in the habit of practicing the other method. Whoever tries this way will never return to the other. Students are almost invariably taught to place patients upon their backs, or sides, and make examinations from the front, and not one in a dozen *ever reaches the os* until after they have been long in practice. They should be taught to make it the other way, and then they would know what it was like before they got into practice, and would immediately recognize it by the touch.

(*c*) *Passing of the Female Catheter.*—When from any cause it becomes necessary to pass the female catheter, much delicacy as well as skill and patience is required, and if possible, ocular inspection should be avoided, and the instrument introduced by touch alone, under the dress or bed-clothes. The accomplishment of the above fact is well nigh impossible at times, even to the most experienced, while the young doctor in his first attempt is almost sure to get in a perspiration before he experiences the gratification of feeling the instrument slip into the patient's urethra. All this trouble comes from following the universal direction, which directs that the finger be moved in the mesial line until it touches the urethral orifice, which will be felt as a slight surrounding elevation, with a center depression. The direction would be well enough, but for the trouble in *finding* said "slight elevation with center depression," without the aid of the eyes. Now a plan much easier than the above, is to introduce the finger into the vagina, upon the anterior wall of which will be felt a small but distinct ridge, the urethra; and by moving downwards upon that ridge with the finger the orifice is easily reached and the catheter directly introduced. Any one, whether experienced or otherwise, by following the above directions, can accomplish the operation without either trouble or waste of time.

(*d*) *Introduction of Pessaries.*—This operation is almost always performed with the patient upon her back, and the failure of many pessaries in the hands of operators is not so much the fault of the instrument, as of the person applying it. With a woman upon her back it requires not a little knack to properly adjust a support or pessary without inflicting upon the patient unnecessary pain, as the weight of the uterus in that position makes it naturally gravitate towards the back, and it must be lifted up before a pessary can be placed; no easy matter in such a posiiion, and often imperfectly accomplished, to the *patient's cost.*

Now the adjusting of a pessary or supporter of any kind should always be done with the patient in *Sims' position* upon *her side* with *knees drawn up*, or in the *knee-breast* position; for the reason that the uterus is most accessible in these positions, and that the air being admitted by the retraction of the posterior vaginal wall, and perineum, distends the vagina, and keeps the uterus in the normal position by the air pressure while the instrument is being placed. One thing more should always be kept in mind when choosing or placing a pessary or supporter, *i. e.*, that none of the uterine ligaments exert a particle of influence in preventing sinking down of the uterus into the pelvis; the uterus may even protrude at the vulva, and yet none of its ligaments be upon the stretch. The only support to that *organ upward is the vagina*, and the retentive power of the abdominal cavity—that power perhaps furnished by the influence of respiration upon the diaphragm, which operates as a tight valve to the abdominal cavity. The only office of the uterine ligaments is to prevent that organ from tipping over; they act exactly like the guy ropes in hoisting a liberty pole—who for a moment would contend that *they* would prevent the pole from sinking down endways if there was no resistance offered by the ground? —*Medical Summary*.

---

### *NEUROTOMY OPTICO-CILIARIS—A NEW OPERATION IN OPHTHALMIC SURGERY.*

BY C. J. LUNDY, M.D.,
Ophthalmic and Aural Surgeon to St. Mary Free Eye and Ear Infirmary, etc.

Within the past year we have occasionally heard something, on this side of the Atlantic, of a new operation for the relief of sympathetic eye troubles—an operation which, in many cases, it would be desirable to substitute for enucleation. That this operation has been but little practiced here, may be judged from the fact that, as yet, none of our special journals contain anything on the subject, and, so far as I am aware, Dr. Chisolm, of Baltimore, is the only one in this country who has written anything concerning it.

Any means which will enable us to avoid the mutilation of a patient (and the removal of a good-looking eye is a mutilation), and which will at the same time afford him immunity from sympathetic mischief in the fellow eye, deserves and should receive a fair trial.

Many times it is desirable to remove an eye for the cosmetic effect alone, and in such cases the operation of neurotomy optico-ciliaris is not indicated. There are many instances, however, in which an eye presents a good external appearance, but at the same time its presence is a source of danger to its fellow, and in such cases the section of the optic and ciliary nerves may and should be substituted for enucleation.

The operation which I am about to describe is, so far as I am aware, the first of the kind which has been performed in this part of the country, and I trust the description, with the notes of a case, may prove of interest.

Theresa Labadie, aged 20, came to consult me on Oct. 20th, and gave the following history:

Nearly two years ago an ulcer formed upon the left cornea, which perforated that tunic, permitting escape of the aqueous humor and prolapse of the iris. From that time till the present she has had more or less irritation, pain and inflammation in the eye, and occasionally an outbreak of a severe character. Marked ciliary injection, slight pain, reduced ocular tension and ciliary tenderness are present, and indicate the existence of a chronic irido-cyclitis. Almost the entire pupillary margin is attached to the cornea at the site of the former ulcer, and the eye has only perception of light. For several months she has suffered from sympathetic irritation of the fellow eye, but for the past two months the sympathetic trouble has been of a more aggravated character. For nearly two weeks the right eye has given her so much trouble she fears total blindness.

Upon examining the right eye, a small ulcer of the cornea is observed, which makes it impossible to determine how much of the trouble is due to sympathetic irritation. Under treatment the ulcer healed kindly in about one week. At the end of the second week there was still present irritation in the right eye, and I advised an operation as the only sure means of getting rid of the trouble and insuring the safety of the fellow eye. About ten days later, or more than three weeks after first consultation, she obtained permission to have an operation performed. In the meantime the irritation in the right eye had become more severe, and while she rejected enucleation she was willing to submit to any other operation which would save her "from blindness."

On November 13th, the patient being under chloroform, I operated as follows:

An incision was made through the conjunctiva and subconjunctival tissue one-eighth of an inch from the sclero-corneal junction and over the insertion of the internal rectus muscle. With a strabismus hook the muscle was caught up and a silk thread was passed through it in order to keep the muscle within easy reach after its division. The tendon was now divided within a line of its ocular attachment, and the conjunctiva was freely dissected up from the sclerotic with the scissors. The ocular end of the tendon was now seized with the forceps, in the hands of an assistant, and the eyeball was rotated forcibly outward towards the temple, by which means the optic nerve, ciliary nerves and vessels were brought well to the nasal side. A long, well-curved enucleation scissors was then introduced close to the eyeball, and carried around to its posterior pole, and the optic nerve and ciliary nerves and blood vessels, which enter the eye at the back, were entirely and completely severed. Immediately after section of the nerves and blood vessels the eye budged forward nearly out of the orbit, and a free escape of blood followed the withdrawal of the scissors. The flow of blood was much increased by the straining of the patient while vomiting. The eye was now turned back into place, the cut ends of the internal rectus tendon were stitched firmly together, a compress bandage was firmly applied, and frequent applications ofcold water were ordered.

A marked degree of exop hthalmos was present, and, to say the least the cosmetic effect was not very imposing. After recovery from the chloroform, she complained of much pain, and morphine, in ¼ grain doses, was ordered. Visiting the patient five hours later, I found her suffering considerable pain and complaining much of nausea. Changed the dressing and used dry lint and compress bandage, and ordered morphine, q. s., to relieve pain. Next day she was much easier, but not entirely free from pain. Lids and conjunctiva considerably swollen, and under-lid echymosed. Exophthalmos not diminished.

November 15th (second day after operation), she is quite free from pain, and slept well during the past night. Chemosis increased, and discoloration extending down upon the cheek, which is somewhat swollen.

November 16th, condition much the same. Some secretion of a muco-purulent character. Ordered greased cloth with slight compress and allowed her the use of the right eye, which has been tied up since operation.

November 17th, ciliary injection of right eye has disappeared, and bright light no longer causes any trouble. No pain in either eye. Chemosis still very great, particularly about the sutures, which evidently cause much irritation.

November 20th, removed sutures from internal rectus. Chemosis and exophthalmos much reduced; echymosis and swelling of underlid and cheek much diminished; motions of the eye tolerably good in all directions; slight diverging squint, probably due to swelling at inner angle of the eye; no pain in either eye.

November 24th, patient came to my office for the first time since the operation. The swelling of the conjunctiva and the exophthalmos are fast disappearing, and motions of the eye are natural in all respects. Instillations of eserine sulphate were begun, with a view of hastening the removal of conjunctival swelling and promoting absorption of the extravasated blood through its action on the vessels. From this date improvement went on steadily, the eye continuing free from pain, and the right remaining perfectly free from all irritation.

December 6th, exophthalmos and swelling of conjunctiva have entirely disappeared, the eye is quite straight, and, save some conjunctival hyperæmia, nothing remains to indicate that any operation had been performed.

The operation has been a complete success, inasmuch as it has fully met the indications for its performance, and leaves the patient free from the dangers which threatened her with total blindness.

Time will enable us to judge more fully and correctly of the merits of this operation, for there is a possibility that the ends of the divided ciliary nerves may unite again, thereby establishing a sympathetic connection which might expose the patient to the dangers which threatened her previous to the operation. This union has occurred after section of other nerves, but on account of the pressing apart, and more or less bruising and turning aside of the ends of the nerves by the large blood clot, which must have formed at the time of operation, it is not probable that direct union of the nerves will ever become established.

By substituting this operation for enucleation, a good-looking eye may often be saved, and the patient may be relieved of the annoyance, trouble and expense of an artificial eye, which, however good it may be, will prove a poor substitute for a natural-looking one, capable of motion in unison with its fellow.—*Mich. Med. News.*

---

WHEN a death occurs in Fiji, it has to be registered; and the native scribes not unfrequently fill the blank left for "cause of death" with the words "medicine supplied by the missionaries."

